# Buffalo Medical and Surgical Association

**Published:** 1856-05

**Authors:** 


"*Buffalo Medical and Surgical Association.*"—At the April meeting of the Buffalo Medical Association, no business of interest to the profession at large was transacted. The Association terminated its existence, and reorganized as a chartered corporation, under the title of the "Buffalo Medica and Surgical Association."

The following Officers, who are also Trustees of the property of the association, were elected for the ensuing year:

| | |
|---|---|
| Dr. SANDFORD EASTMAN, - - | President. |
| " AUSTIN FLINT, - - - - | Vice-president. |
| " SANFORD B. HUNT, - - - | Secretary. |
| " JAMES M. NEWMAN, - - - | Treasurer. |
| " WM. HOWELL, - - - | Librarian. |

The President elect, Dr. Eastman, being absent, Dr. Flint was conducted to the rostrum by a committee of three, (Drs. White, Wyckoff and Mixer,) and was greeted with cordial applause as he took the chair.

